# Lactate Metabolism, Signaling, and Function in Brain Development, Synaptic Plasticity, Angiogenesis, and Neurodegenerative Diseases

**DOI:** 10.3390/ijms241713398

**Published:** 2023-08-29

**Authors:** Anika Wu, Daehoon Lee, Wen-Cheng Xiong

**Affiliations:** 1 Department of Neurosciences, School of Medicine, Case Western Reserve University, Cleveland, OH 44106, USA; axw368@case.edu (A.W.); dxl660@case.edu (D.L.); 2 Louis Stokes Cleveland Veterans Affairs Medical Center, Cleveland, OH 44106, USA

**Keywords:** energy, lactate, development, synaptic plasticity, neurodegeneration, dysmetabolism, neuroprotective

## Abstract

Neural tissue requires a great metabolic demand despite negligible intrinsic energy stores. As a result, the central nervous system (CNS) depends upon a continuous influx of metabolic substrates from the blood. Disruption of this process can lead to impairment of neurological functions, loss of consciousness, and coma within minutes. Intricate neurovascular networks permit both spatially and temporally appropriate metabolic substrate delivery. Lactate is the end product of anaerobic or aerobic glycolysis, converted from pyruvate by lactate dehydrogenase-5 (LDH-5). Although abundant in the brain, it was traditionally considered a byproduct or waste of glycolysis. However, recent evidence indicates lactate may be an important energy source as well as a metabolic signaling molecule for the brain and astrocytes—the most abundant glial cell—playing a crucial role in energy delivery, storage, production, and utilization. The astrocyte–neuron lactate-shuttle hypothesis states that lactate, once released into the extracellular space by astrocytes, can be up-taken and metabolized by neurons. This review focuses on this hypothesis, highlighting lactate’s emerging role in the brain, with particular emphasis on its role during development, synaptic plasticity, angiogenesis, and disease.

## 1. Introduction

The brain consumes 20% of the body’s energy and oxygen uptake despite representing only 2% of the body’s weight [1]. Within the brain, neurons are estimated to consume nearly 80% of the brain’s allocated energy stores, while glial cells, such as astrocytes, microglia, and oligodendrocytes, utilize a much smaller amount of energy [2]. The energy is used for numerous critical tasks, including restoring neuronal membrane potentials after depolarization, axoplasmic transport, vesicle recycling, and neurotransmitter synthesis [3,4,5]. As such, energy requirements are not uniformly distributed but region-specific and dependent on neuronal activity. Thus, the regulation of cellular energy metabolism and metabolite supply is critical in maintaining normal brain/neuron function, as energy consumption is highly demanded and dynamic [2].

Glucose is considered to be the primary substrate fueling oxidative metabolism and neural activity [6,7]. Glucose is transported to neural tissue through the capillaries, entering cells through specialized glucose transporters (GLUTs) [8]. Blood-borne glucose transport across the blood–brain barrier occurs through the glucose transporter 1 (GLUT1), whereas glucose uptake in neurons and astrocytes is regulated by GLUT3 and GLUT1, respectively [8]. Interestingly, PET imaging indicates a possible uncoupling between glucose utilization and oxygen consumption [9,10]. This emerging information has led to alternative substrates, such as lactate, a substrate typically regarded as a toxic metabolic waste product, being considered to possibly sustain neuronal activity [11]. Lactate concentration is similar to that of glucose in the extracellular space [12], and astrocytes and neurons are able to both oxidize glucose and lactate but use different metabolic pathways to do so. Though differing, the metabolic profiles of glucose and lactate are complementary, leading to metabolic cooperativity. First proposed by Pellerin and Magisarrati, the astrocyte–neuron lactate-shuttle Hypothesis (ANLSH) highlights the astrocyte’s active role in neuronal energy consumption; while lactate was previously seen solely as a waste product, this hypothesis suggests this “byproduct” may play a larger role [13]. Astrocytes have a higher rate of glycolysis in comparison to neurons therefore allowing astrocytes to readily provide a continuous supply of energy to the brain [6]. Glucose is predominantly taken up in astrocytes and anaerobically metabolized into lactate [14]. Lactate possesses distinctive attributes that render it crucial for neurons during neuronal events such as depolarization. The energy demand required for neuronal depolarization is not sufficient and cannot be adequately fulfilled solely on conventional glucose supply since glucose utilization necessitates the initiation of glycolysis, a time-sensitive and relatively inefficient energy generation cascade. Additionally, this solution requires a consistent presence of glucose or for glucose to be stored in an accessible form. However, neurons lack the capacity to maintain such a reservoir [15]. In contrast, astrocytes are capable of harboring energy-rich metabolites and store the majority of brain glycogen which can be readily used [16]. Upon neuronal activation, glycogen is converted into lactate within astrocytes and subsequently transferred to neurons to be converted into pyruvate, further facilitating energy production [17]. Recent studies have shown neurons to have a slower glycolytic rate due to their inability to upregulate a pathway necessary to produce the activator of a glycolytic enzyme in neurons known as phosphofrucokinase-1 (PFK1) during increased activity and cellular stress. In the same study, overexpression of Pfkfb3 to activate neuronal glycolysis led to apoptosis, therefore, suggesting neurons are unable to sustain high glycolytic rates, further emphasizing the importance of astrocytes regarding maintaining the steep neuronal metabolic demands [18]. In essence, astrocytes function as energy reservoirs that are mobilized when required.

**Figure 1:**
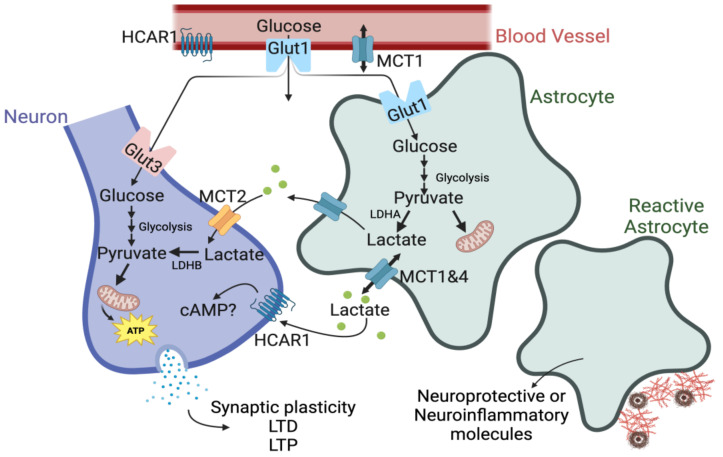
Astrocyte–neuron lactate-shuttle Hypothesis. First described by Pellerin and Magistretti in 1994 [13], ANLS proposed that, during neuronal activity, glutamate is released into the synaptic cleft of neurons and glucose from blood vessels, which is then taken up by astrocytes. Upon entering astrocytes, glucose is converted into pyruvate via glycolysis. Pyruvate is then converted into lactate by the enzyme lactate dehydrogenase isoenzyme A (LDHA). Lactate is then transferred out of astrocytes by MCT1/4 into the extracellular space, and neuronal MCT2 is able to uptake lactate for aerobic utilization to support cellular processes critical for maintaining brain homeostasis. Abbreviations: cAMP: Cyclic adenosine monophosphate; GLUT: Glucose transporter; HCAR1: Hydroxycarboxylic acid receptor 1; LDH: Lactate dehydrogenase; MCT: Monocarboxylate transporter; LTD: Long-term depression; LTP: Long-term potentiation.

In several experiments, glial cells were found to uptake significant amounts of glucose fluorescent analogs in cerebellar slices [14,19]. Similarly, approximately half of brain glucose uptake is perpetuated by astrocytes in resting rat brains [20,21]. However, recent research has also demonstrated that the brain uses lactate during hypoglycemia or when lactate levels in the blood are high [22]. In vitro studies have shown that in the absence of glucose, lactate can support neural activity in the brain, and lactate, but not glucose, can recover neurons in hypoxic conditions [23]. Once astrocytic lactate is produced, it is transferred to, and used by, active neurons. Recently, in vivo experiments have shown a lactate gradient between astrocytes and neurons, supporting the ANLSH [24]. Lactate is transported into neurons via monocarboxylate transporters (MCTs)—protein-linked membrane carriers that shuttle monocarboxylates, such as lactate—to fuel the activity-related energy demands of neurons. MCT1 is expressed primarily in astrocytes, endothelial cells of micro-vessels, ependymocytes, and oligodendrocytes [25,26,27,28]. MCT2, on the other hand, is found primarily in neurons, while MCT4 is expressed nearly exclusively by astrocytes [28]. The ANLSH shifts the focal point of the primary neuronal energy substrate from glucose toward lactates derived from astrocytes (Figure 1 and Table 1).

It is important to note that despite the growing body of evidence in support for the ANLSH, there is research that casts doubt upon and criticizes the hypothesis. One study provided in vitro evidence of neurons being able to directly metabolize glucose in the absence of astrocytic lactate, suggesting astrocytes may not be as important as previously thought [29]. From the same group, using genetically encoded fluorescent biosensors to measure neuronal metabolic responses to stimulation in awake mice, it was found that neurons directly consume glucose, providing further evidence against the ANLSH [30]. Some of the main criticisms of the ANLSH include conflicting evidence as to where lactate originates from, how much lactate is produced by astrocytes, and how lactate concentration returns to baseline is not clear. Several studies have shown lactate as a source of energy during stressful conditions, such as injury or stress, but the energy substrate used in resting conditions is hotly debated [31,32,33]. Though acknowledged as a source of energy, some research has shown that in physiological conditions, lactate is not the preferred energy source for neurons [29,30]. Further research is needed to understand lactate’s role in the brain and direct experimental observation of lactate from astrocytes to neighboring neurons to strengthen the ANLSH.

Despite conflicting reports, there is little doubt that astrocytes release lactate and that lactate is crucial for development and is emerging as a key metabolite for synaptic plasticity and CNS pathologies of the aging brain. It modulates cognitive function primarily through two mechanisms, as an energy substrate to energetically demanding neurons due to increased synaptic activity and as signaling molecule to initiate plasticity-related singling transduction pathways [34].

**Table 1:** Key components of ANLS substrate specificity, expression, and lactate affinity.

Key Components of ANLS	Gene Name	Role	Expression in the Brain	Lactate Affinity	
MCT1	*SLC16A1*	Transporters of lactate, pyruvate, acetoacetate, β-hydroxybutyrate, XP13512, and GHB	Endothelial cells of microvessels, astrocytes, ependymocytes, oligodendrocytes	3.5–10 mM	[27,28,35]
MCT2	*SLC16A7*	Transporters of lactate and pyruvate	Neurons	0.5–0.75 mM	[35,36]
MCT4	*SLC16A3*	Transporters of lactate, pyruvate, acetoacetate, and β-hydroxybutyrate	Astrocytes	22–28 mM	[35,37]
GLUT1	*SLC2A1*	Transporters of glucose, galactose, mannose, glucosamine, and ascorbic acid	Astrocytes, endothelial cells		[8,38]
GLUT3	*SLC2A3*	Transporters of glucose, mannose, galactose, and xylose	Neurons		[8,38]
HCAR1	*HCAR1/GPR81*	Lactate receptor	pial fibroblast-like cells that line the vessels, pericyte-like cells along intracerebral microvessels, neurons	Lactate activates HCAR1 in a range of 1–20 mM	[39,40,41]

## 2. Lactate Metabolism and Signaling in Development

Neural energy metabolism during development differs from that of adults, as lactate is the main substrate of the developing brain during the perinatal period [42]. The use of glucose is only 10% of the adult value primarily due to low levels of glucose transporters, which must be acquired via breastfeeding [42]. In rodents, lactate is used by the brain in fetal, early newborn, and suckling rats [43]. Lactate’s role is especially critical for the brain during the postnatal period as the brain is continuing to develop [44]. Continuous supply of metabolic substrates is required to sustain brain development. During the perinatal period, nutrients (i.e., glucose, lactate, amino acids, and fatty acids) are passed from the mother through the transplacental passage [45,46]. Lactate can be transported to the fetus from the mother via the placental membrane and is most concentrated in the placenta leading to its accumulation in the blood during late gestation. Coinciding with the increased lactate concentrations during late gestation, MCT activity is increased. MCT expression begins during mid-gestation and is largely increased during the late fetal and neonatal period [47]. Lactate found in fetal blood is rapidly metabolized after delivery. Early in vitro experiments showed that cultured astrocytes and neurons from neonates utilize lactate significantly more than other metabolites, such as glucose, 3-hydroxbutyrate, or glutamine [48]. Glucose availability is scarce during the postnatal period, but lactate is constantly supplied from the blood to both neurons and astrocytes for use during brain development [49].

Lactate’s role during development has not been well studied, but it is known to have a neuroprotective effect in adults. Recent research, through the lens of hypoxic-ischemic (HI) encephalopathy, has studied its role in rat neonates. The removal of lactate after HI insults in neonates produces more brain damage, while the addition of glucose had no additive effect. Multiple interparietal injections of lactate following HI insult led to full recovery of long-term memory, sensorimotor abilities and neurological reflexes [50,51]. Lactate can act as a signaling molecule, and recent studies have shown that lactate can bind to a receptor called HCAR1 (hydroxycarboxylic acid receptor), previously referred to as GPR81 (G-protein-coupled receptor 81), in human and rodent brains [52,53]. Following HI, newborn mice with or without (partial or complete loss of) the gene coding for HCAR1 were compared. It was found that the complete loss of HCAR1 led to the downregulation of regeneration in neuronal progenitor cells and glial cells and impaired microglia activation, but partial loss led to some recovery in the insulted brain [52]. Together, this suggests that lactate plays an essential role for cell survival and recovery in the developing brain.

## 3. Lactate Metabolism and Signaling in Synaptic Plasticity

Lactate clearly plays a crucial role in brain development and function and a number of different processes, including synaptic plasticity. Blood-derived lactate is a critical energy substrate during development to meet neuronal metabolic demands, but in the adult brain, lactate used by neurons is thought to come from astrocytes. The ANLS is capable of supplying metabolic demands in response to enhanced synaptic activity and participating in the process of plasticity. The brain at a resting state is estimated to have a low-millimolar range of extracellular levels of lactate [54], but during physical exercise lactate levels can increase up to 10–20 mM [55]. There is increasing evidence of lactate’s role in learning and memory, suggesting a close relationship between synaptic activity and lactate [56,57]. During synaptic activity, lactate levels are reported to increase two-fold [58], and well-known mediators of plasticity processes, such as synaptogenesis, experience-dependent synaptic remodeling, and synaptic efficacy, are glutamate receptor N-Methyl-D-aspartate receptors (NMDARs) [59]. Lactate works to enhance calcium influx and inward current flow once NDMARs activity is induced by glutamate and glycine. Here, lactate acts as a signaling molecule, and NDMARs and downstream extracellular signal-regulated kinase (ERK1/2) signaling are activated in response to elevated calcium levels leading to increased expression of genes implicated in synaptic plasticity in neurons. Several genes involved in activity maintenance and neuronal plasticity have been identified, such as Arc, BDNF, c-Fos, and Zif268 [56,57,60] (Table 2). When using NMDAR antagonist MK801, the effect of lactate on the genes was abolished, therefore implying the activation of the genes is dependent on NDMAR activity [57,60].

Recent evidence has also shown that HCAR1 is present in neurons and can regulate neuronal activity [41,61]. Receptor activation leads to the down regulation of cyclic adenosine 3′,5′ monophosphate (cAMP), of GABAergic, and principal neurons in the hippocampus in vitro [41]. cAMP works as intracellular second messenger that is strongly implicated in specific forms of hippocampal dependent memory by targeting kinases such as cAMP-dependent kinases, like PKA. The cAMP/PKA system is implicated in spatial memory and contextual conditioning, and PKA can target several key proteins, such as NMDA, involved in synaptic plasticity and memory storage [62]. Another proposed mechanism of HCAR1 modulation of neuronal activity is thought to be through the activation of G_α_ and G_βγ_ subunits [63]. Activation of HCAR1 resulted in decreased firing frequency and neuronal excitability. This was further supported in another study using seizure models, where neurons have excessive continuous bursts of action potentials and prolonged depolarization. Mice lacking HCAR1 were more susceptible to developing longer and more severe seizures in comparison to WT mice. Lactate perfusion to activate HCAR1 was able to reduce activity of CA1 neurons and inhibit excitatory transmission in WT but not in HCAR1 deficient mice [61].

Lactate has been shown to be necessary for the maintenance of long-term potentiation (LTP) in vivo experiments in the rodent hippocampus. Several studies have shown that inhibition of astrocytic lactate produced via glycogenolysis can impair memory processes in rodents. In DAB treated P1 chicks, memory consolidation was interrupted and reduced glycogen content was found during aversive memory training [64]. Bilaterally injecting DAB into rats blocked long term inhibitory memory in avoidance tasks and long-term potentiation (LTP) [65]. This coincides with downregulated plasticity-related molecules Arc, p-CFL1, and p-CREB [66]. The effects of the inhibitor can be rescued with lactate. This was similarly seen when reducing MCT1 or MCT4 in the hippocampus which resulted in the disruption of long-term memory formation and hippocampal neurogenesis [67]. However, when inhibiting MCT2 expression, glucose or lactate is not able to rescue cognitive impairment, implying that neuronal MCT2 expression is required for LTP and long-term memory (LTM) formation. MCTs have also been reported to be required in contextual fear memory acquisition in the amygdala. MCT inhibitor 4-CIN expression in fear contextual conditioning training significantly reduced freezing time [68]. Long-term memory formation in mice is thought to depend on synaptic plasticity and neuronal activity-dependent genes. These studies suggest lactate as the main substrate to fuel neuronal responses necessary for long-term memory. The studies also highlight the crucial role of MCTs for lactate transportation into neurons to regulate synaptic activity, potentially allowing MCTs to be possible drug targets in neurodegenerative diseases where synaptic activity is altered.

Alternatively, lactate can indirectly regulate synaptic plasticity by modulating the activity of microglia, which are capable of adapting their metabolic status. Single cell RNA-seq datasets have shown that microglia have the metabolic capability to utilize different bioenergetic substrates such as lactate [69,70]. Increasing evidence has shown microglia’s capiblity to produce lactate through glycolsis and to import lactate from the extracellular space [70,71]. In addition, recent reports have shown proper machinery, such as MCT expression in microglia alluding lactate, to be a novel bioenergetic substrate to modulate cellular functions such as synaptic plasticity [72,73]. These emerging studies provide evidence supporting a microglia–neuron lactate shuttle. Lactate is also shown to play a role in neuronal myelination which would support and allow for properly functioning neurons [74,75].

## 4. Lactate Metabolism and Signaling in Brain Angiogenesis

Angiogenesis is a highly regulated process of forming new blood vessels from pre-existing microvasculature. This biological process plays a critical role in developing and maintaining the brain’s function under exercise and wound repair conditions [76]. There has been increasing evidence of a positive correlation between cerebral perfusion and cognition [77]. Interestingly, angiogenesis in the brain is also regulated by multiple brain cells, including neurons, astrocytes, and microglia [78,79,80]. Notably, lactate has been proposed to modulate angiogenesis by acting on the lactate receptor HCAR1 due to its wide distribution and concentrations in pericyte like cells on intracerebral microvessels and pial fibroblast like cells of blood vessels supplying blood to the brain [40]. During intense exercise, it is well known that blood lactate levels increase by several folds, and lactate accumulated in the blood is able to pass through the blood–brain barrier (BBB) via MCTs [6,7]. In one study, HCAR1 was been found to mediate exercise-induced brain vascularization stimulated by vascular endothelial growth factor A (VEGFA) [40]. The angiogenetic factor is also able to directly enhance synaptic function and neurogenesis. In HCAR1 deficient mice, vascularization commonly found after exercise or induced by subcutaneously injected lactate did not lead to VEGFA activation in comparison to wildtype mice [40]. The mechanism underlying HCAR1 activation to VEGFA production is unclear. Several pathways like that of synaptic plasticity, such as ERK1/2, which was seen in wildtype mice but not HCAR1 KO mice, in the hippocampus, are thought to be involved in this event. Further investigations are necessary to understand the underlying mechanisms, as HCAR1 may be a target for therapeutic effects in neurological diseases, where cerebral vasculature is impaired, thus, leading to deficient energy substrates provided due to its link to cerebral angiogenesis. Additionally, the functions of MCTs in lactate or exercise-induced vasculature in the brain remain unknown. Further research is also necessary to address this issue.

## 5. Lactate Metabolism and Signaling in Diseases

Abnormal metabolism, such as mitochondrial dysfunction and defective glucose metabolism and/or neuronal glucose uptake, is commonly found in neurodegenerative diseases, such as Alzheimer disease (AD), Parkinson disease (PD), amyotrophic lateral sclerosis (ALS), and Huntington disease (HD) [81,82]. Reactive astrocytes are associated with neurodegenerative diseases, such as AD and PD, and can secrete molecules into the extracellular space that can have neuroprotective or neurodegenerative functions [83]. Specifically in AD, which is characterized by amyloid beta plaques and neurofibrillary tangles, glucose uptake is decreased while increased levels of lactate are found in the cerebrospinal fluid (CSF) [84,85], which is thought to be due to pro-inflammatory microglia [86,87]. Interestingly, in the 3xTg AD mouse model, astrocytic lactate production through aerobic glycolysis was decreased [88]. Like AD patients, PD patients, who have severe dopaminergic loss, have elevated CSF lactate levels [89,90]. While in the MPTP-induced mouse model of PD, researchers found upregulated hexokinase 2 increased lactate, which prompted the degeneration of dopaminergic neurons [89]. Further studies are urgently needed as lactate levels are clearly altered, though there are differing results across studies, as lactate can be used as a biomarker to clinically diagnose patients with neurodegenerative diseases by measuring CSF levels.

It has also been found that the impairment of neural energy metabolism due to defective lactate shuttling from glial cells to neurons can lead to neuronal loss and degeneration similar to that of AD [91]. In neurodegenerative diseases, it has been well characterized that astrocytes are dysfunctional and can lead to an impairment of lactate production [92]. In turn this can lead to a decrease in neuronal energy levels and progression of the disease. Patients with cognitive impairments were found to have reduced LDH5 activity, an enzyme critical for glucose conversion to lactate, and GLUT1 and GLUT3 expression, both important components in the ANLS [93,94,95]. Young adults carrying ApoE4, a genetic risk factor for late-onset AD, are found to have increased levels of MCT2 and decreased levels of MCT4 in the posterior cingulate of the limbic system [96,97]. Interestingly, this does not correspond with animal studies, but this may be due to low sample numbers and analyzation of protein and RNA levels in a broad scope as opposed to single cell sequencing. Several AD rodent models show neuronal MCT2 and EAAT1/2 levels in astrocytes to be significantly reduced [98,99]. Similarly, rats receiving hippocampal injections of amyloid beta peptide fragments showed decreased MCT2 expression, as well as decreased lactate and memory and learning deficits [98]. Application of a MCT2 inhibitor, 4-CIN, to prevent lactate uptake in hippocampal neurons increased glutamate-induced neuronal discharge abnormalities in the CA1 region of the hippocampus as is frequently seen in AD [100]. Together these findings highlight the dysfunction of key components in ANLS as potential pathological mechanisms in AD.

Aside from AD, there have been advancements in understanding lactate’s role in the pathogenesis of other cognitive disorders. For example, ketamine, a dissociative anesthetic that acts on the CNS through the antagonism of NMDARs, has been shown to cause dose-dependent impairment to episodic and working memory if administered long term; alterations in MCT protein expression (increases in MCT1 and 4, but decrease in MCT2) are observed alongside behavioral changes, suggesting the observed memory changes may be related to aberrant hippocampal MCT expression [67]. The knockdown of astrocytic MCT4 in the motor cortex led to decreased neuronal activity in the motor cortex and known associated regions such as the dorsal striatum and ventral thalamus [101]. Additionally, motor performance, learning, dendritic spines, and plasticity-related protein expression were significantly reduced. As previously mentioned, lactate plays a critical role in activating synaptic plasticity-related genes in response to neural activity through the NMDAR to start a signaling cascade to regulate ERK1/2 [57]. Memory and addiction are thought to share the same circuitry and molecular mechanisms therefore leading researchers to conclude the astrocyte to neuron lactate transfer is involved in storage and retrieval of addictive drug-related memories [102]. The administration of DAB, an inhibitor of glycogen phosphorylase, was sufficient to impair cocaine induced conditioned place preference in rats [102]. These findings show that the abnormal expression of key components involved in the ANLS, such as metabolic enzymes and transports, affect the lactate shuttle, thus leading to negative outcomes, such as exacerbating neurodegenerative-like phenotypes. As a result, key components of ANLS are of great interest as novel therapeutic targets in neurological disorders, including AD, PD, and addiction.

L-Lactate has also been established as a neuroprotectant during injury in both human and animal models [103]. In the context of ischemic stroke, mice that received permanent middle cerebral artery occlusion treated with lactate and recombinant tissue plasminogen activator, the only approved drug for treatment, experienced a reduction in lesion size, and an improved neurological outcome was found [104]. Stroke leads to excitotoxicity, a process that results in neuronal damage or death due to the excessive stimulation of NDMARs, causing an overload of intracellular calcium, thus triggering downstream neurotoxic cascades. Excitotoxicity has been proposed to play a role in neurodegenerative diseases, and lactate, as a signaling molecule, can be a neuroprotectant against toxic insults through the activation of P2Y2, thus activating the intracellular neuroprotective signaling pathway [105] Lactate has also been proposed to work as a neuroprotectant by lowering pH levels during its conversion to lactatic acid within neurons. The slight decrease in pH in turn facilitates the firing of action potentials and enhance neuronal excitability therefore making the depolarization of neurons more efficient [106]. The improved excitability may have neuroprotective effects through the maintenance of proper neuronal signaling. In the context of neurodegenerative diseases commonly associated with neuroinflammation, lactate is thought to influence inflammation and contribute to anti-inflammatory responses in several proposed mechanisms, such as pH modulation, regulation of immune cell function, metabolic reprogramming, anti-oxidative effects, and microglial regulation [107]. Lactate plays a crucial role in injury and disease, and some researchers have suggested the consumption of alcohol in moderation, though not a viable treatment option due to its risk of addiction, since it is the greatest possible source of lactate from our daily diet [108]. It is important to note though that lactate’s effect as a neuroprotectant and its anti-inflammatory effect is evolving, and more research is needed to understand the mechanisms involved in order for lactate to be harnessed as a potential therapeutic.

## 6. Concluding Remarks and Future Perspectives

The conventional hypothesis of the central nervous system’s metabolism asserted glucose to be the primary substrate during neural activity, but increasing evidence has challenged this idea. The astrocyte–neuron lactate-shuttle hypothesis proposes a mechanism and a source of energy, lactate, to fuel high energy demands to maintain brain homeostasis throughout an organism’s life. The ANLS plays a critical role in important brain functions, such as development, synaptic plasticity, angiogenesis, and disease states. An impairment in the shuttling of lactate due to decreased levels of key players leads to cognitive decline, such as AD, and given the tight link between metabolic dysfunction and neurodegenerative disease, it warrants further studies. The hypothesis also recognizes and highlights lactate’s significant role in a number of physiological and pathological processes, such as the regulation of energy metabolism, memory formation, and recovery after injury, which are regulated by lactate as either a metabolic substrate or as a signaling molecule. Currently, lactate is used as an indicator for disease diagnoses, such as cancer, but it can be used as a potential therapeutic in diseases where energy metabolism is altered or as a neuroprotectant against insults to the brain.

## Figures and Tables

**Table 2:** Genes affected by lactate. Several genes involved in synaptic plasticity, neuroprotection, and cell death are regulated by lactate and identified in cortical neurons by transcriptome analysis [56]. In neurodegenerative states, energy metabolism is altered and increased cell death is observed. These are genes of interest that may be affected due to impairment of the astrocyte to neuron lactate shuttle.

Genes Upregulated by Lactate	Genes Downregulated by Lactate
Synaptic Plasticity	Cell Death
Arc	
Bdnf	
c-Fos	
Zif268	
Atf4	Txnip
Nr4a1	Apafl
Gadd45b	Bcl2111
Gadd45g	Hrk
Map3k11	
Dusp4	
Dusp6	
Dusp10	
**Neuroprotection**	
Bdnf	
Grfa2	
Nr4a2	
Vegfa	

## References

[1] Raichle M.E., Gusnard D.A. (2002). Appraising the Brain’s Energy Budget. Proc. Natl. Acad. Sci. USA.

[2] Hyder F., Rothman D.L., Bennett M.R. (2013). Cortical Energy Demands of Signaling and Nonsignaling Components in Brain Are Conserved across Mammalian Species and Activity Levels. Proc. Natl. Acad. Sci. USA.

[3] Attwell D., Laughlin S.B. (2001). An Energy Budget for Signaling in the Grey Matter of the Brain. J. Cereb. Blood Flow Metab..

[4] Harris J.J., Jolivet R., Attwell D. (2012). Synaptic Energy Use and Supply. Neuron.

[5] Rangaraju V., Calloway N., Ryan T.A. (2014). Activity-Driven Local ATP Synthesis Is Required for Synaptic Function. Cell.

[6] Hertz L., Peng L., Dienel G.A. (2007). Energy Metabolism in Astrocytes: High Rate of Oxidative Metabolism and Spatiotemporal Dependence on Glycolysis/Glycogenolysis. J. Cereb. Blood Flow Metab..

[7] Dienel G.A. (2012). Brain Lactate Metabolism: The Discoveries and the Controversies. J. Cereb. Blood Flow Metab..

[8] Vannucci S.J., Maher F., Simpson I.A. (1997). Glucose Transporter Proteins in Brain: Delivery of Glucose to Neurons and Glia. Glia.

[9] Fox P.T., Raichle M.E. (1986). Focal Physiological Uncoupling of Cerebral Blood Flow and Oxidative Metabolism during Somatosensory Stimulation in Human Subjects. Proc. Natl. Acad. Sci. USA.

[10] Fox P.T., Raichle M.E., Mintun M.A., Dence C. (1988). Nonoxidative Glucose Consumption during Focal Physiologic Neural Activity. Science.

[11] Mintun M.A., Vlassenko A.G., Rundle M.M., Raichle M.E. (2004). Increased Lactate/Pyruvate Ratio Augments Blood Flow in Physiologically Activated Human Brain. Proc. Natl. Acad. Sci. USA.

[12] Pfeuffer J., Tkác I., Gruetter R. (2000). Extracellular-Intracellular Distribution of Glucose and Lactate in the Rat Brain Assessed Noninvasively by Diffusion-Weighted 1H Nuclear Magnetic Resonance Spectroscopy in Vivo. J. Cereb. Blood Flow Metab..

[13] Pellerin L., Pellegri G., Bittar P.G., Charnay Y., Bouras C., Martin J.L., Stella N., Magistretti P.J. (1998). Evidence Supporting the Existence of an Activity-Dependent Astrocyte-Neuron Lactate Shuttle. Dev. Neurosci..

[14] Chuquet J., Quilichini P., Nimchinsky E.A., Buzsáki G. (2010). Predominant Enhancement of Glucose Uptake in Astrocytes versus Neurons during Activation of the Somatosensory Cortex. J. Neurosci..

[15] Schönfeld P., Reiser G. (2013). Why Does Brain Metabolism Not Favor Burning of Fatty Acids to Provide Energy?—Reflections on Disadvantages of the Use of Free Fatty Acids as Fuel for Brain. J. Cereb. Blood Flow Metab..

[16] Matsui T., Omuro H., Liu Y.-F., Soya M., Shima T., McEwen B.S., Soya H. (2017). Astrocytic Glycogen-Derived Lactate Fuels the Brain during Exhaustive Exercise to Maintain Endurance Capacity. Proc. Natl. Acad. Sci. USA.

[17] Falkowska A., Gutowska I., Goschorska M., Nowacki P., Chlubek D., Baranowska-Bosiacka I. (2015). Energy Metabolism of the Brain, Including the Cooperation between Astrocytes and Neurons, Especially in the Context of Glycogen Metabolism. Int. J. Mol. Sci..

[18] Herrero-Mendez A., Almeida A., Fernández E., Maestre C., Moncada S., Bolaños J.P. (2009). The Bioenergetic and Antioxidant Status of Neurons Is Controlled by Continuous Degradation of a Key Glycolytic Enzyme by APC/C–Cdh1. Nat. Cell Biol..

[19] Barros L.F., San Martín A., Sotelo-Hitschfeld T., Lerchundi R., Fernández-Moncada I., Ruminot I., Gutiérrez R., Valdebenito R., Ceballo S., Alegría K. (2013). Small Is Fast: Astrocytic Glucose and Lactate Metabolism at Cellular Resolution. Front. Cell. Neurosci..

[20] Barros L.F., Courjaret R., Jakoby P., Loaiza A., Lohr C., Deitmer J.W. (2009). Preferential Transport and Metabolism of Glucose in Bergmann Glia over Purkinje Cells: A Multiphoton Study of Cerebellar Slices. Glia.

[21] Nehlig A., Wittendorp-Rechenmann E., Dao Lam C. (2004). Selective Uptake of [14C] 2-Deoxyglucose by Neurons and Astrocytes: High-Resolution Microautoradiographic Imaging by Cellular 14C-Trajectography Combined with Immunohistochemistry. J. Cereb. Blood Flow Metab..

[22] Wiegers E.C., Rooijackers H.M., Tack C.J., Philips B.W., Heerschap A., van der Graaf M., de Galan B.E. (2019). Effect of Lactate Administration on Brain Lactate Levels during Hypoglycemia in Patients with Type 1 Diabetes. J. Cereb. Blood Flow Metab..

[23] Kelleher J.A., Chan P.H., Chan T.Y., Gregory G.A. (1993). Modification of Hypoxia-Induced Injury in Cultured Rat Astrocytes by High Levels of Glucose. Stroke.

[24] Mächler P., Wyss M.T., Elsayed M., Stobart J., Gutierrez R., von Faber-Castell A., Kaelin V., Zuend M., San Martín A., Romero-Gómez I. (2016). In Vivo Evidence for a Lactate Gradient from Astrocytes to Neurons. Cell Metab..

[25] Gerhart D.Z., Enerson B.E., Zhdankina O.Y., Leino R.L., Drewes L.R. (1997). Expression of Monocarboxylate Transporter MCT1 by Brain Endothelium and Glia in Adult and Suckling Rats. Am. J. Physiol..

[26] Hanu R., McKenna M., O’Neill A., Resneck W.G., Bloch R.J. (2000). Monocarboxylic Acid Transporters, MCT1 and MCT2, in Cortical Astrocytes in Vitro and in Vivo. Am. J. Physiol. Cell Physiol..

[27] Pierre K., Pellerin L., Debernardi R., Riederer B.M., Magistretti P.J. (2000). Cell-Specific Localization of Monocarboxylate Transporters, MCT1 and MCT2, in the Adult Mouse Brain Revealed by Double Immunohistochemical Labeling and Confocal Microscopy. Neuroscience.

[28] Pierre K., Pellerin L. (2005). Monocarboxylate Transporters in the Central Nervous System: Distribution, Regulation and Function. J. Neurochem..

[29] Díaz-García C.M., Yellen G. (2019). Neurons Rely on Glucose Rather than Astrocytic Lactate during Stimulation. J. Neurosci. Res..

[30] Díaz-García C.M., Mongeon R., Lahmann C., Koveal D., Zucker H., Yellen G. (2017). Neuronal Stimulation Triggers Neuronal Glycolysis and Not Lactate Uptake. Cell Metab..

[31] Kasischke K.A., Squire L.R. (2009). Activity-Dependent Metabolism in Glia and Neurons. Encyclopedia of Neuroscience.

[32] Dembitskaya Y., Piette C., Perez S., Berry H., Magistretti P.J., Venance L. (2022). Lactate Supply Overtakes Glucose When Neural Computational and Cognitive Loads Scale Up. Proc. Natl. Acad. Sci. USA.

[33] Tauffenberger A., Fiumelli H., Almustafa S., Magistretti P.J. (2019). Lactate and Pyruvate Promote Oxidative Stress Resistance through Hormetic ROS Signaling. Cell Death Dis..

[34] Nalbandian M., Takeda M. (2016). Lactate as a Signaling Molecule That Regulates Exercise-Induced Adaptations. Biology.

[35] Halestrap A.P. (2012). The Monocarboxylate Transporter Family—Structure and Functional Characterization. IUBMB Life.

[36] Bröer S., Bröer A., Schneider H.-P., Stegen C., Halestrap A.P., Deitmer J.W. (1999). Characterization of the High-Affinity Monocarboxylate Transporter MCT2 in Xenopus Laevis Oocytes. Biochem. J..

[37] Dimmer K.-S., FRIEDRICH B., Lang F., Deitmer J.W., BRÖER S. (2000). The Low-Affinity Monocarboxylate Transporter MCT4 Is Adapted to the Export of Lactate in Highly Glycolytic Cells. Biochem. J..

[38] Mueckler M., Thorens B. (2013). The SLC2 (GLUT) Family of Membrane Transporters. Mol. Asp. Med..

[39] Liu C., Wu J., Zhu J., Kuei C., Yu J., Shelton J., Sutton S.W., Li X., Yun S.J., Mirzadegan T. (2009). Lactate Inhibits Lipolysis in Fat Cells through Activation of an Orphan G-Protein-Coupled Receptor, GPR81. J. Biol. Chem..

[40] Morland C., Andersson K.A., Haugen Ø.P., Hadzic A., Kleppa L., Gille A., Rinholm J.E., Palibrk V., Diget E.H., Kennedy L.H. (2017). Exercise Induces Cerebral VEGF and Angiogenesis via the Lactate Receptor HCAR1. Nat. Commun..

[41] Lauritzen K.H., Morland C., Puchades M., Holm-Hansen S., Hagelin E.M., Lauritzen F., Attramadal H., Storm-Mathisen J., Gjedde A., Bergersen L.H. (2014). Lactate Receptor Sites Link Neurotransmission, Neurovascular Coupling, and Brain Energy Metabolism. Cereb. Cortex.

[42] Medina J.M., Tabernero A. (2005). Lactate Utilization by Brain Cells and Its Role in CNS Development. J. Neurosci. Res..

[43] Shambaugh G.E., Mrozak S.C., Freinkel N. (1977). Fetal Fuels. I. Utilization of Ketones by Isolated Tissues at Various Stages of Maturation and Maternal Nutrition during Late Gestation. Metabolism.

[44] Burd L.I., Jones M.D., Simmons M.A., Makowski E.L., Meschia G., Battaglia F.C. (1975). Placental Production and Foetal Utilisation of Lactate and Pyruvate. Nature.

[45] Balkovetz D.F., Leibach F.H., Mahesh V.B., Ganapathy V. (1988). A Proton Gradient Is the Driving Force for Uphill Transport of Lactate in Human Placental Brush-Border Membrane Vesicles. J. Biol. Chem..

[46] Delatorre S.R.A., Serrano M.A., Medina J.M. (1992). Carrier-mediated beta-d-hydroxybutyrate transport in brush-border membrane-vesicles from rat placenta. Pediatr. Res..

[47] Girard J.R., Ferré P., Gilbert M., Kervran A., Assan R., Marliss E.B. (1977). Fetal Metabolic Response to Maternal Fasting in the Rat. Am. J. Physiol.-Endocrinol. Metab..

[48] Vicario C., Tabernero A., Medina J.M. (1993). Regulation of Lactate Metabolism by Albumin in Rat Neurons and Astrocytes from Primary Culture. Pediatr. Res..

[49] Juanes M.C., Arizmendi C., Medina J.M. (1986). Attenuation of Postnatal Hypoxia in the Premature Newborn Rat by Maternal Treatment with Dexamethasone: Its Relationship with Lung Phospholipid Content. Neonatology.

[50] Allen K.A., Brandon D.H. (2011). Hypoxic Ischemic Encephalopathy: Pathophysiology and Experimental Treatments. Newborn Infant Nurs. Rev..

[51] Roumes H., Dumont U., Sanchez S., Mazuel L., Blanc J., Raffard G., Chateil J.F., Pellerin L., Bouzier-Sore A.K. (2021). Neuroprotective role of lactate in rat neonatal hypoxia-ischemia. J. Cereb. Blood Flow Metab. Off. J. Int. Soc. Cereb. Blood Flow Metab..

[52] Kennedy L., Glesaaen E.R., Palibrk V., Pannone M., Wang W., Al-Jabri A., Suganthan R., Meyer N., Austbø M.L., Lin X. (2022). Lactate Receptor HCAR1 Regulates Neurogenesis and Microglia Activation after Neonatal Hypoxia-Ischemia. eLife.

[53] Briquet M., Rocher A.B., Alessandri M., Rosenberg N., de Castro Abrantes H., Wellbourne-Wood J., Schmuziger C., Ginet V., Puyal J., Pralong E. (2022). Activation of Lactate Receptor HCAR1 Down-Modulates Neuronal Activity in Rodent and Human Brain Tissue. J. Cereb. Blood Flow Metab..

[54] Abi-Saab W.M., Maggs D.G., Jones T., Jacob R., Srihari V., Thompson J., Kerr D., Leone P., Krystal J.H., Spencer D.D. (2002). Striking Differences in Glucose and Lactate Levels between Brain Extracellular Fluid and Plasma in Conscious Human Subjects: Effects of Hyperglycemia and Hypoglycemia. J. Cereb. Blood Flow Metab..

[55] Offermanns S. (2017). Hydroxy-Carboxylic Acid Receptor Actions in Metabolism. Trends Endocrinol. Metab..

[56] Margineanu M.B., Mahmood H., Fiumelli H., Magistretti P.J. (2018). L-Lactate Regulates the Expression of Synaptic Plasticity and Neuroprotection Genes in Cortical Neurons: A Transcriptome Analysis. Front. Mol. Neurosci..

[57] Yang J., Ruchti E., Petit J.-M., Jourdain P., Grenningloh G., Allaman I., Magistretti P.J. (2014). Lactate Promotes Plasticity Gene Expression by Potentiating NMDA Signaling in Neurons. Proc. Natl. Acad. Sci. USA.

[58] Dienel G.A., Ball K.K., Cruz N.F. (2007). A Glycogen Phosphorylase Inhibitor Selectively Enhances Local Rates of Glucose Utilization in Brain during Sensory Stimulation of Conscious Rats: Implications for Glycogen Turnover. J. Neurochem..

[59] Quinlan E.M., Philpot B.D., Huganir R.L., Bear M.F. (1999). Rapid, Experience-Dependent Expression of Synaptic NMDA Receptors in Visual Cortex in Vivo. Nat. Neurosci..

[60] Bajaffer A., Mineta K., Magistretti P., Gojobori T. (2022). Lactate-Mediated Neural Plasticity Genes Emerged during the Evolution of Memory Systems. Sci. Rep..

[61] Skwarzynska D., Sun H., Williamson J., Kasprzak I., Kapur J. (2022). Glycolysis Regulates Neuronal Excitability via Lactate Receptor, HCA1R. Brain.

[62] Abel T., Nguyen P.V., Barad M., Deuel T.A.S., Kandel E.R., Bourtchouladze R. (1997). Genetic Demonstration of a Role for PKA in the Late Phase of LTP and in Hippocampus-Based Long-Term Memory. Cell.

[63] de Castro Abrantes H., Briquet M., Schmuziger C., Restivo L., Puyal J., Rosenberg N., Rocher A.B., Offermanns S., Chatton J.Y. (2019). The Lactate Receptor HCAR1 Modulates Neuronal Network Activity through the Activation of G(α) and G(Βγ) Subunits. J. Neurosci..

[64] Gibbs M.E., Anderson D.G., Hertz L. (2006). Inhibition of Glycogenolysis in Astrocytes Interrupts Memory Consolidation in Young Chickens. Glia.

[65] Suzuki A., Stern S.A., Bozdagi O., Huntley G.W., Walker R.H., Magistretti P.J., Alberini C.M. (2011). Astrocyte-Neuron Lactate Transport Is Required for Long-Term Memory Formation. Cell.

[66] Xue X., Liu B., Hu J., Bian X., Lou S. (2022). The Potential Mechanisms of Lactate in Mediating Exercise-Enhanced Cognitive Function: A Dual Role as an Energy Supply Substrate and a Signaling Molecule. Nutr. Metab. Lond..

[67] Ding R., Tan Y., Du A., Wen G., Ren X., Yao H., Ren W., Liu H., Wang X., Yu H. (2020). Redistribution of Monocarboxylate 1 and 4 in Hippocampus and Spatial Memory Impairment Induced by Long-Term Ketamine Administration. Front. Behav. Neurosci..

[68] Kong L., Zhao Y., Zhou W.J., Yu H., Teng S.W., Guo Q., Chen Z., Wang Y. (2017). Direct Neuronal Glucose Uptake Is Required for Contextual Fear Acquisition in the Dorsal Hippocampus. Front. Mol. Neurosci..

[69] Monsorno K., Buckinx A., Paolicelli R.C. (2022). Microglial Metabolic Flexibility: Emerging Roles for Lactate. Trends Endocrinol. Metab..

[70] Nagy A.M., Fekete R., Horvath G., Koncsos G., Kriston C., Sebestyen A., Giricz Z., Kornyei Z., Madarasz E., Tretter L. (2018). Versatility of Microglial Bioenergetic Machinery under Starving Conditions. Biochim. Biophys. Acta BBA—Bioenerg..

[71] Nair S., Sobotka K.S., Joshi P., Gressens P., Fleiss B., Thornton C., Mallard C., Hagberg H. (2019). Lipopolysaccharide-induced Alteration of Mitochondrial Morphology Induces a Metabolic Shift in Microglia Modulating the Inflammatory Response in Vitro and in Vivo. Glia.

[72] Zhang Y., Chen K., Sloan S.A., Bennett M.L., Scholze A.R., O’Keeffe S., Phatnani H.P., Guarnieri P., Caneda C., Ruderisch N. (2014). An RNA-Sequencing Transcriptome and Splicing Database of Glia, Neurons, and Vascular Cells of the Cerebral Cortex. J. Neurosci..

[73] Hammond T.R., Dufort C., Dissing-Olesen L., Giera S., Young A., Wysoker A., Walker A.J., Gergits F., Segel M., Nemesh J. (2019). Single-Cell RNA Sequencing of Microglia throughout the Mouse Lifespan and in the Injured Brain Reveals Complex Cell-State Changes. Immunity.

[74] Stadelmann C., Timmler S., Barrantes-Freer A. (2019). Mikael Simons Myelin in the Central Nervous System: Structure, Function, and Pathology. Physiol. Rev..

[75] Rinholm J.E., Hamilton N.B., Kessaris N., Richardson W.D., Bergersen L.H., Attwell D. (2011). Regulation of Oligodendrocyte Development and Myelination by Glucose and Lactate. J. Neurosci..

[76] Zhang Y., Wang H., Oliveira R.H.M., Zhao C., Popel A.S. (2022). Systems Biology of Angiogenesis Signaling: Computational Models and Omics. WIREs Mech. Dis..

[77] Wightman E.L., Haskell-Ramsay C.F., Thompson K.G., Blackwell J.R., Winyard P.G., Forster J., Jones A.M., Kennedy D.O. (2015). Dietary Nitrate Modulates Cerebral Blood Flow Parameters and Cognitive Performance in Humans: A Double-Blind, Placebo-Controlled, Crossover Investigation. Physiol. Behav..

[78] Zhao Y., Tang F., Lee D., Xiong W.-C. (2021). Expression of Low Level of VPS35-MCherry Fusion Protein Diminishes Vps35 Depletion Induced Neuron Terminal Differentiation Deficits and Neurodegenerative Pathology, and Prevents Neonatal Death. Int. J. Mol. Sci..

[79] Yao L.-L., Hu J.-X., Li Q., Lee D., Ren X., Zhang J.-S., Sun D., Zhang H.-S., Wang Y.-G., Mei L. (2020). Astrocytic Neogenin/Netrin-1 Pathway Promotes Blood Vessel Homeostasis and Function in Mouse Cortex. J. Clin. Investig..

[80] Dudiki T., Meller J., Mahajan G., Liu H., Zhevlakova I., Stefl S., Witherow C., Podrez E., Kothapalli C.R., Byzova T.V. (2020). Microglia Control Vascular Architecture via a TGFβ1 Dependent Paracrine Mechanism Linked to Tissue Mechanics. Nat. Commun..

[81] Han R., Liang J., Zhou B. (2021). Glucose Metabolic Dysfunction in Neurodegenerative Diseases-New Mechanistic Insights and the Potential of Hypoxia as a Prospective Therapy Targeting Metabolic Reprogramming. Int. J. Mol. Sci..

[82] Cai H., Cong W.N., Ji S., Rothman S., Maudsley S., Martin B. (2012). Metabolic Dysfunction in Alzheimer’s Disease and Related Neurodegenerative Disorders. Curr. Alzheimer Res..

[83] Li K., Li J., Zheng J., Qin S. (2019). Reactive Astrocytes in Neurodegenerative Diseases. Aging Dis..

[84] Xiang X., Wind K., Wiedemann T., Blume T., Shi Y., Briel N., Beyer L., Biechele G., Eckenweber F., Zatcepin A. (2021). Microglial Activation States Drive Glucose Uptake and FDG-PET Alterations in Neurodegenerative Diseases. Sci. Transl. Med..

[85] Liguori C., Chiaravalloti A., Sancesario G., Stefani A., Sancesario G.M., Mercuri N.B., Schillaci O., Pierantozzi M. (2016). Cerebrospinal Fluid Lactate Levels and Brain [18F]FDG PET Hypometabolism within the Default Mode Network in Alzheimer’s Disease. Eur. J. Nucl. Med. Mol. Imaging.

[86] Boland B., Yu W.H., Corti O., Mollereau B., Henriques A., Bezard E., Pastores G.M., Rubinsztein D.C., Nixon R.A., Duchen M.R. (2018). Promoting the Clearance of Neurotoxic Proteins in Neurodegenerative Disorders of Ageing. Nat. Rev. Drug Discov..

[87] Aldana B.I. (2019). Microglia-Specific Metabolic Changes in Neurodegeneration. J. Mol. Biol..

[88] Le Douce J., Maugard M., Veran J., Matos M., Jégo P., Vigneron P.-A., Faivre E., Toussay X., Vandenberghe M., Balbastre Y. (2020). Impairment of Glycolysis-Derived l-Serine Production in Astrocytes Contributes to Cognitive Deficits in Alzheimer’s Disease. Cell Metab..

[89] Li J., Chen L., Qin Q., Wang D., Zhao J., Gao H., Yuan X., Zhang J., Zou Y., Mao Z. (2022). Upregulated Hexokinase 2 Expression Induces the Apoptosis of Dopaminergic Neurons by Promoting Lactate Production in Parkinson’s Disease. Neurobiol. Dis..

[90] Liguori C., Stefani A., Fernandes M., Cerroni R., Mercuri N.B., Pierantozzi M. (2022). Biomarkers of Cerebral Glucose Metabolism and Neurodegeneration in Parkinson’s Disease: A Cerebrospinal Fluid-Based Study. J. Park. Dis..

[91] Sun Y., Wang Y., Chen S.-T., Chen Y.-J., Shen J., Yao W.-B., Gao X.-D., Chen S. (2020). Modulation of the Astrocyte-Neuron Lactate Shuttle System Contributes to Neuroprotective Action of Fibroblast Growth Factor 21. Theranostics.

[92] Phatnani H., Maniatis T. (2015). Astrocytes in Neurodegenerative Disease. Cold Spring Harb. Perspect. Biol..

[93] Simpson I.A., Chundu K.R., Davies-Hill T., Honer W.G., Davies P. (1994). Decreased Concentrations of GLUT1 and GLUT3 Glucose Transporters in the Brains of Patients with Alzheimer’s Disease. Ann. Neurol. Off. J. Am. Neurol. Assoc. Child Neurol. Soc..

[94] Harr S.D., Simonian N.A., Hyman B.T. (1995). Functional Alterations in Alzheimer’s Disease: Decreased Glucose Transporter 3 Immunoreactivity in the Perforant Pathway Terminal Zone. J. Neuropathol. Exp. Neurol..

[95] Mooradian A.D., Chung H.C., Shah G.N. (1997). GLUT-1 Expression in the Cerebra of Patients with Alzheimer’s Disease. Neurobiol. Aging.

[96] Smith C.J., Ashford J.W., Perfetti T.A. (2019). Putative Survival Advantages in Young Apolipoprotein Ɛ4 Carriers Are Associated with Increased Neural Stress. J. Alzheimers Dis..

[97] Perkins M., Wolf A.B., Chavira B., Shonebarger D., Meckel J.P., Leung L., Ballina L., Ly S., Saini A., Jones T.B. (2016). Altered Energy Metabolism Pathways in the Posterior Cingulate in Young Adult Apolipoprotein E Ɛ4 Carriers. J. Alzheimers Dis..

[98] Lu W., Huang J., Sun S., Huang S., Gan S., Xu J., Yang M., Xu S., Jiang X. (2015). Changes in Lactate Content and Monocarboxylate Transporter 2 Expression in Aβ 25-35-Treated Rat Model of Alzheimer’s Disease. Neurol. Sci..

[99] Schallier A., Smolders I., Van Dam D., Loyens E., De Deyn P.P., Michotte A., Michotte Y., Massie A. (2011). Region-and Age-Specific Changes in Glutamate Transport in the AβPP23 Mouse Model for Alzheimer’s Disease. J. Alzheimers Dis..

[100] Schurr A., Miller J.J., Payne R.S., Rigor B.M. (1999). An Increase in Lactate Output by Brain Tissue Serves to Meet the Energy Needs of Glutamate-Activated Neurons. J. Neurosci..

[101] Lundquist A.J., Llewellyn G.N., Kishi S.H., Jakowec N.A., Cannon P.M., Petzinger G.M., Jakowec M.W. (2021). Knockdown of Astrocytic Monocarboxylate Transporter 4 (MCT4) in the Motor Cortex Leads to Loss of Dendritic Spines and a Deficit in Motor Learning. bioRxiv.

[102] Boury-Jamot B., Carrard A., Martin J.L., Halfon O., Magistretti P.J., Boutrel B. (2016). Disrupting Astrocyte-Neuron Lactate Transfer Persistently Reduces Conditioned Responses to Cocaine. Mol. Psychiatry.

[103] Patet C., Suys T., Carteron L., Oddo M. (2016). Cerebral Lactate Metabolism after Traumatic Brain Injury. Curr. Neurol. Neurosci. Rep..

[104] Buscemi L., Blochet C., Price M., Magistretti P.J., Lei H., Hirt L. (2020). Extended Preclinical Investigation of Lactate for Neuroprotection after Ischemic Stroke. Clin. Transl. Neurosci..

[105] Jourdain P., Allaman I., Rothenfusser K., Fiumelli H., Marquet P., Magistretti P.J. (2016). L-Lactate Protects Neurons against Excitotoxicity: Implication of an ATP-Mediated Signaling Cascade. Sci. Rep..

[106] Ruffin V.A., Salameh A.I., Boron W.F., Parker M.D. (2014). Intracellular PH Regulation by Acid-Base Transporters in Mammalian Neurons. Front. Physiol..

[107] Caslin H.L., Abebayehu D., Pinette J.A., Ryan J.J. (2021). Lactate Is a Metabolic Mediator That Shapes Immune Cell Fate and Function. Front. Physiol..

[108] Collins M.A., Neafsey E.J., Mukamal K.J., Gray M.O., Parks D.A., Das D.K., Korthuis R.J. (2009). Alcohol in Moderation, Cardioprotection, and Neuroprotection: Epidemiological Considerations and Mechanistic Studies. Alcohol. Clin. Exp. Res..

